# Supplementary material for: A neuronal network of mitochondrial dynamics regulates metastasis
Source: Nat Commun. 2016 Dec 19;7:13730. doi: 10.1038/ncomms13730 (PMC5187409; doi:10.1038/ncomms13730)
Supplement: Supplementary Information — Supplementary Figures and Supplementary Tables. [file ncomms13730-s1.pdf]

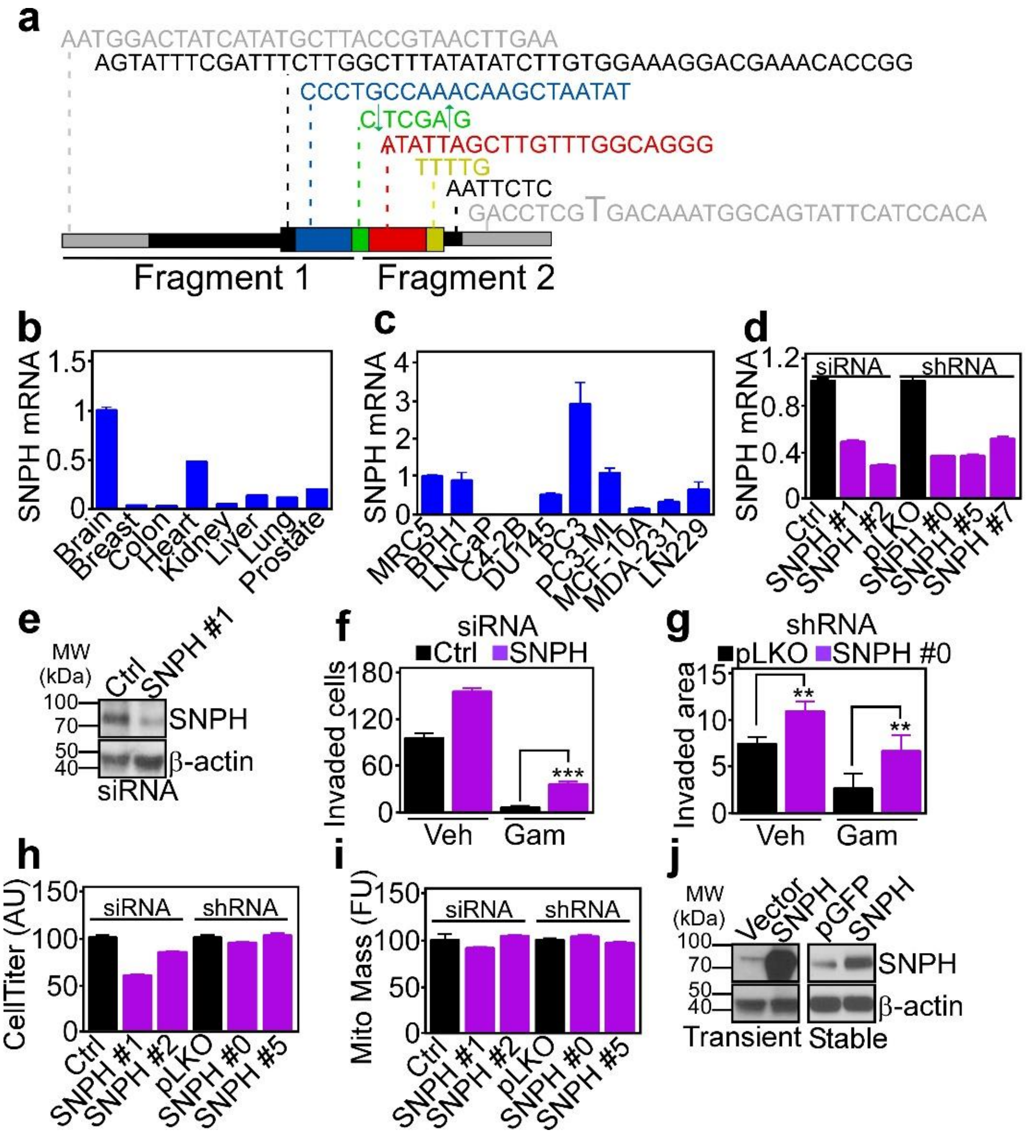

Supplementary Figure 1. **shRNA screening for mitochondrial regulators of prostate cancer metastasis.** (**a**) Two replicate samples from the shRNA screening were processed by NGS with generation of 99,983,355 and 98,756,442 50 bp quality reads. A full product of 176-178 bp in length is indicated. The two fragments generated by *Xho*I digestion of the hairpins are indicated. Gray, PCR primers for amplification of shRNA-containing product; **Black bold**, 1 bp mismatch with the lentivirus sequence to avoid the creation of a second *Xho*I site; **Thick bar**, unique shRNA insert; Blue, sense mature shRNA sequence; Red, antisense mature shRNA sequence; Green, hairpin loop, targeted by the indicated *Xho*I excision; Yellow, variable insert sequence as annotated in the TCR library. (**b,c**) RNA samples isolated from the indicated human normal tissues (**b**) or cultured cell lines (**c**) were subject to PCR amplification with SNPH-directed primers and normalized to β-actin mRNA. Data is represented as mean ± s.e.m. (n=3). (**d**) PC3 cells were transfected with the indicated siRNA or transduced with shRNA and analyzed for SNPH mRNA expression by quantitative PCR. Data is represented as mean ± s.e.m. (n=3). (**e**) PC3 cells were transfected with control siRNA (Ctrl) or SNPH-directed siRNA and analyzed by Western blotting. (**f,g**) PC3 cells were transfected with control siRNA or SNPH-directed siRNA (**f**) or stably transduced with pLKO or SNPH-directed shRNAs (#0) (**g**) and analyzed for Matrigel invasion in the presence or absence of Gamitrinib (Gam 5 μM, 16 h). Data are represented as mean ± s.e.m. (n=3). **, p<0.01; ***, p<0.0001. (**h**) Cell proliferation was measured with a CellTiter assay on identical conditions as the invasion assays from (f,g). Data represents the mean ± s.e.m. (n=3). (**i**) PC3 cells were labeled with Mitotracker green FM and mitochondrial mass was measured by fluorometry and normalized to total cell number. Data represents the mean ± s.e.m. (n=3). (**j**) PC3 cells were transiently (*left*) or stably (*right*) transfected with vector, pGFP or SNPH cDNA, and analyzed by Western blotting.

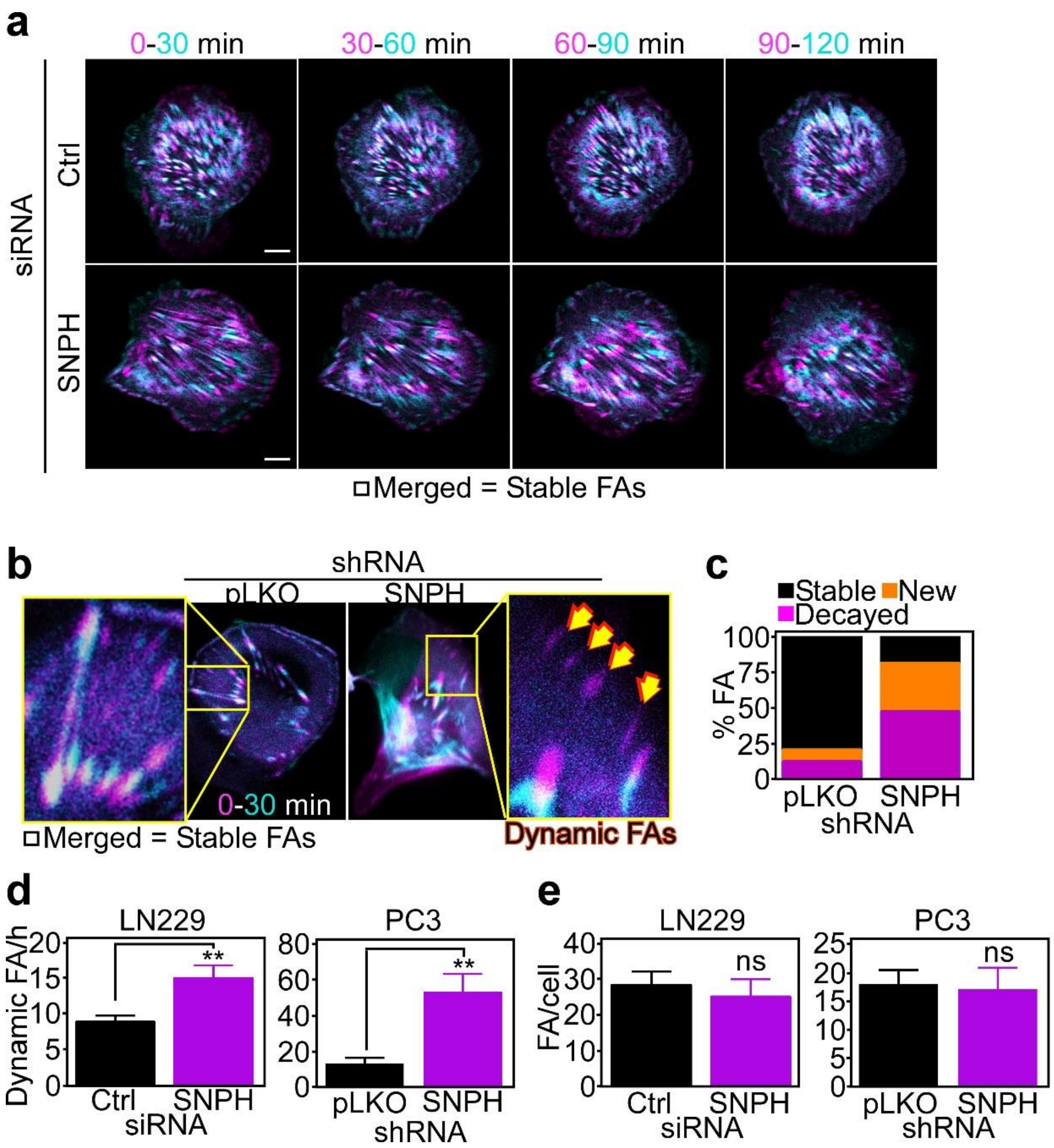

Supplementary Figure 2. **SNPH regulation of Focal Adhesion (FA) dynamics.** Cells were transfected with siRNA, transduced with Talin-RFP Bac-Man virus to label FA complexes and analyzed by time-lapse videomicroscopy throughout the indicated time intervals. (**a**) Successive 30 min intervals are shown for two representative LN229 cells. FA, focal adhesions. Scale bar, 10 µm. (**b**) FA from two representative PC3 cells are shown. Arrows, dynamic FAs. (**c,d**) FA complexes were manually counted and classified as stable (mature, stable sliding) or dynamic (decaying, newly formed). (**c**) Distribution of FAs from PC3 cells (n=205 FAs per group). (**d**) Rate of formation and disassembly of FA per hour. Data is represented as mean ± s.e.m. (n=10 cells). **, p=0.0063-0.0014. (**e**) Total number of FA per cell was represented as mean ± s.e.m. (n=10 cells). ns, not significant.

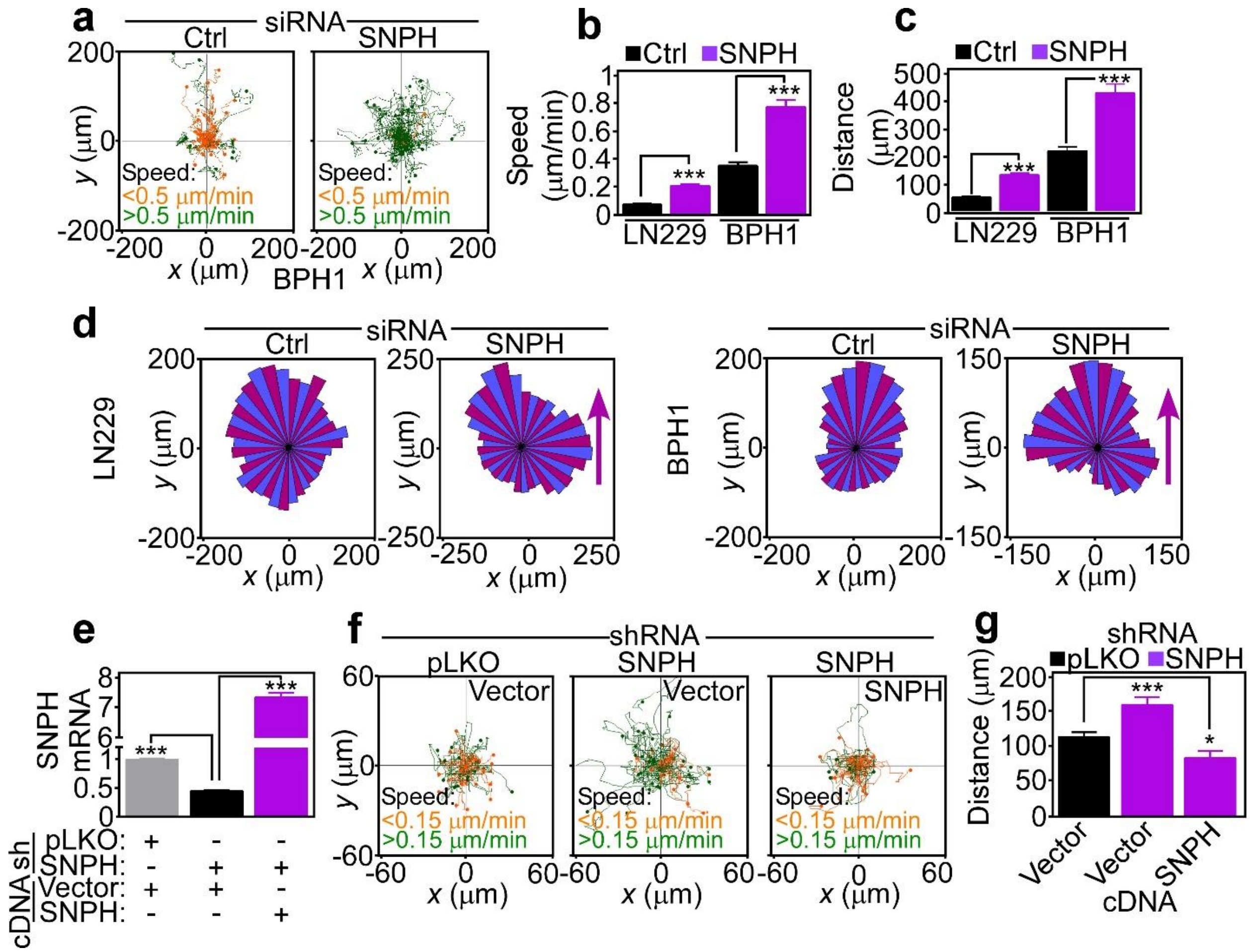

Supplementary Figure 3. **SNPH regulation of tumor chemotaxis.** (**a**) BPH1 cells transfected with control siRNA or SNPH-directed siRNA were analyzed by time-lapse videomicroscopy in a 2D chemotaxis chamber and the trajectories of cell migration were analyzed. The cutoff velocities are indicated. Each tracing corresponds to an individual cell. n(Ctrl)=61, n(SNPH)=46. (**b,c**) siRNA-transfected LN229 or BPH1 cells were quantified for speed of cell migration (**b**), and total distance traveled by individual cells (**c**). Data are represented as mean ± s.e.m. Sample sizes for BPH1 were as in (a). Sample size for LN229 were n(Ctrl)=60, n(SNPH)=89. ***, p<0.001-0.0001. (**d**) Rose plot analysis of directional 2D chemotaxis in tumor cells transfected with the indicated siRNA. Arrow, direction of chemotactic gradient. *Left*, LN229; *right*, BPH1 cells. (**e**) PC3 cells transduced with pLKO or SNPH-directed shRNA (#0) were reconstituted with vector or SNPH cDNA followed by quantification of SNPH mRNA levels by qPCR. Data is represented as mean ± s.e.m. (n=3). ***, p<0.001. (**f,g**) The experimental conditions are as in (e) except that transduced PC3 cells reconstituted with vector or SNPH cDNA were seeded for 2D chemotaxis experiments. Individual cell trajectories and cutoff velocities are represented in 2D plots (**f**), and the cumulative distance traveled (**g**) was calculated. Data is represented as mean ± s.e.m. n(pLKO+vector)=46, n(SNPHsh+vector)=48, n(SNPHsh+SNPHcDNA)=46. *, p=0.0209; ***, p=0.0007.

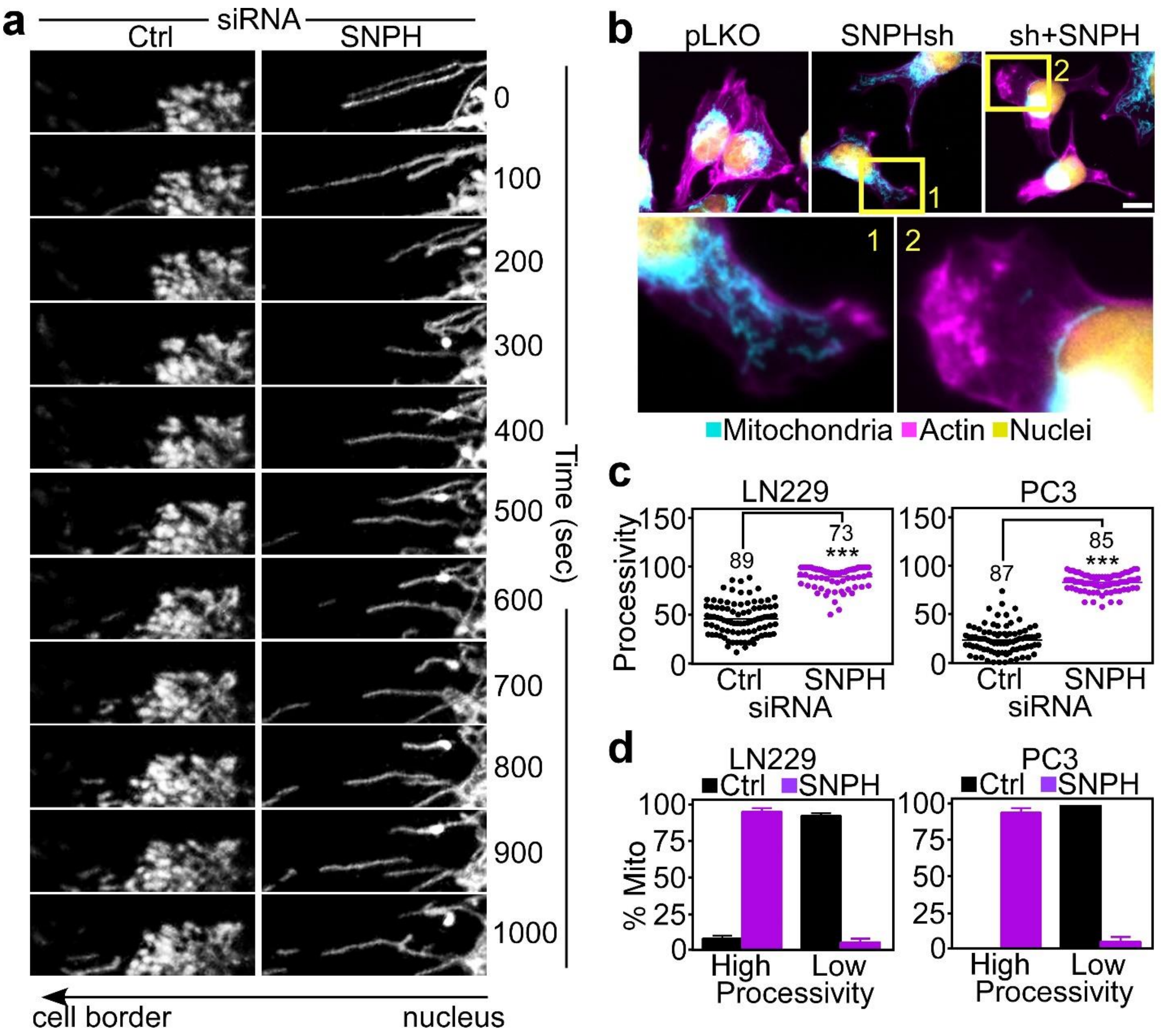

Supplementary Figure 4. **SNPH regulation of mitochondrial trafficking.** (**a**) LN229 cells transfected with control siRNA or SNPH-directed siRNA and expressing Mito-RFP were analyzed by time-lapse videomicroscopy at the indicated time intervals. Representative images are shown. (**b**) PC3 cells stably expressing a control (pLKO) or SNPH-targeting shRNA #0 were transfected with vector or SNPH cDNA. Representative fluorescence images are shown. Scale bar, 10 µm. Zoomed panels show that enhanced mitochondrial trafficking to the cell border (1) in SNPHsh cells is prevented by reconstitution with SNPH cDNA (2). (**c**) Quantification of mitochondrial processivity (time mitochondria spend in motion relative to the total time of tracks) from the experiment in (a). Each point represents an individual mitochondrion. Sample size is indicated in the graph. ***, $p<0.0001$. (**d**) Distribution of *high-processivity* (>70% of time spent in motion) and *low processivity* (<70% of time spent in motion) mitochondria in cells transfected with the indicated siRNA. Data is represented as mean ± s.e.m (n=10).

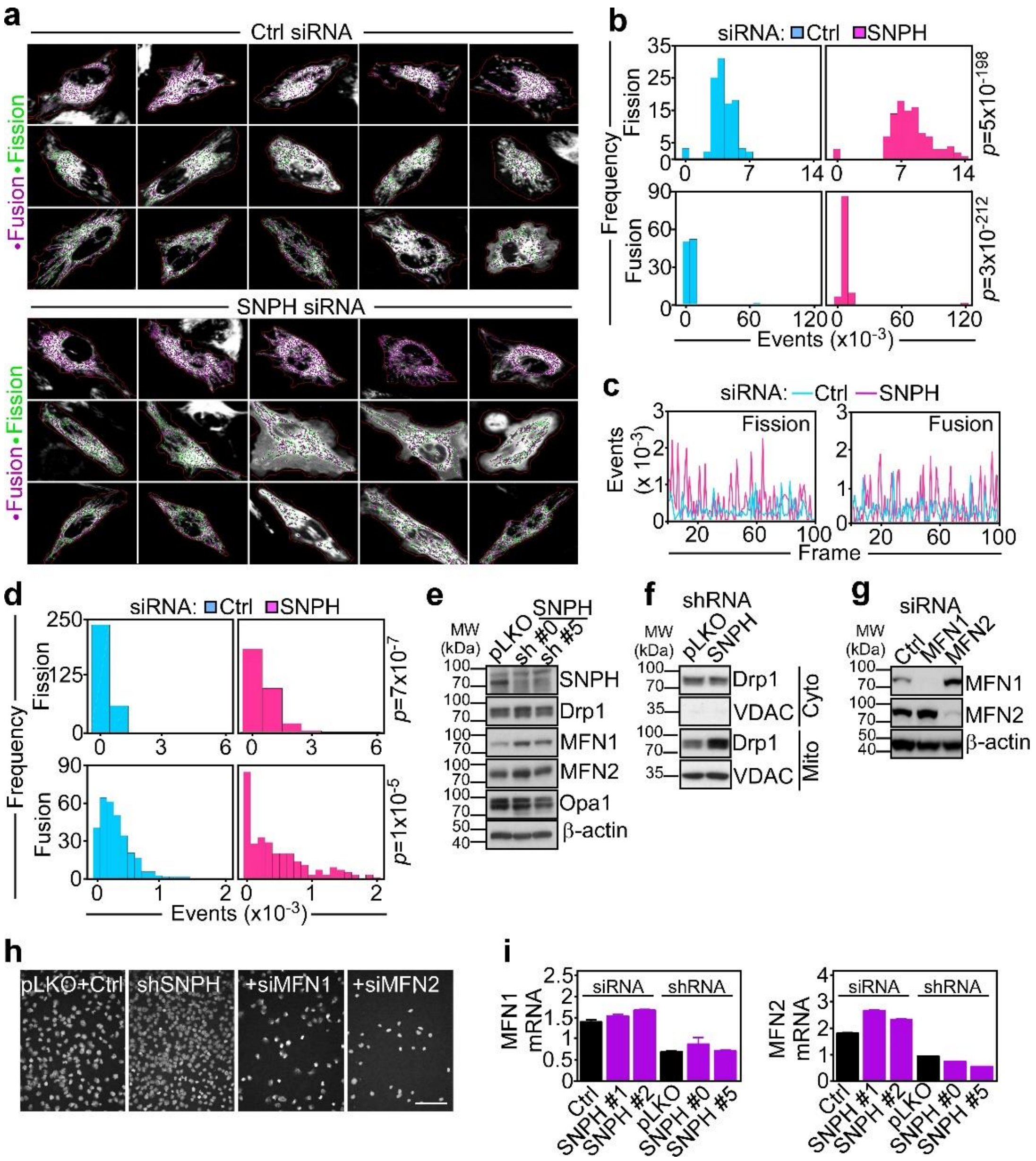

Supplementary Figure 5. **SNPH regulation of mitochondrial dynamics.** (**a-d**) Cells transfected with control siRNA (Ctrl) or SNPH-directed siRNA were labeled with Mito-RFP and analyzed by time lapse microscopy. (**a**) Projections of time-lapse mitochondrial images for individual LN229 cells were overlaid with fission and fusion events from all time points. (**b**) Computational analysis of fusion and fission events in LN229 cells. Distributions of normalized events per frame were compared with two sample Kolmogorov-Smirnov test and p values are indicated. (**c**) Mitochondrial fission and fusion events in PC3 cells. The first 100 frames (100 seconds) of time lapse are represented. (**d**) Computational analysis of fusion and fission events in PC3 cells. Distributions of events per frame were compared with two sample Kolmogorov-Smirnov test and p values are indicated. (**e**) PC3 cells transduced with pLKO or the indicated SNPH-directed shRNAs were analyzed by Western blotting. (**f**) Mitochondrial (Mito) and cytosolic (Cyto) fractions were analyzed by Western blotting. (**g**) PC3 cells were transfected with control (Ctrl) Mfn1 or Mfn2 siRNA and analyzed by Western blotting. (**h**) PC3 cells from (g) were analyzed for Matrigel invasion. Representative micrographs of DAPI-stained nuclei of invaded cells are shown. Scale bar, 200 μm. (**i**) PC3 cells were analyzed by qPCR to quantitate the levels of Mfn1 and Mfn2. Expression was normalized to β-actin mRNA levels. Data are represented as mean ± s.e.m. (n=3).

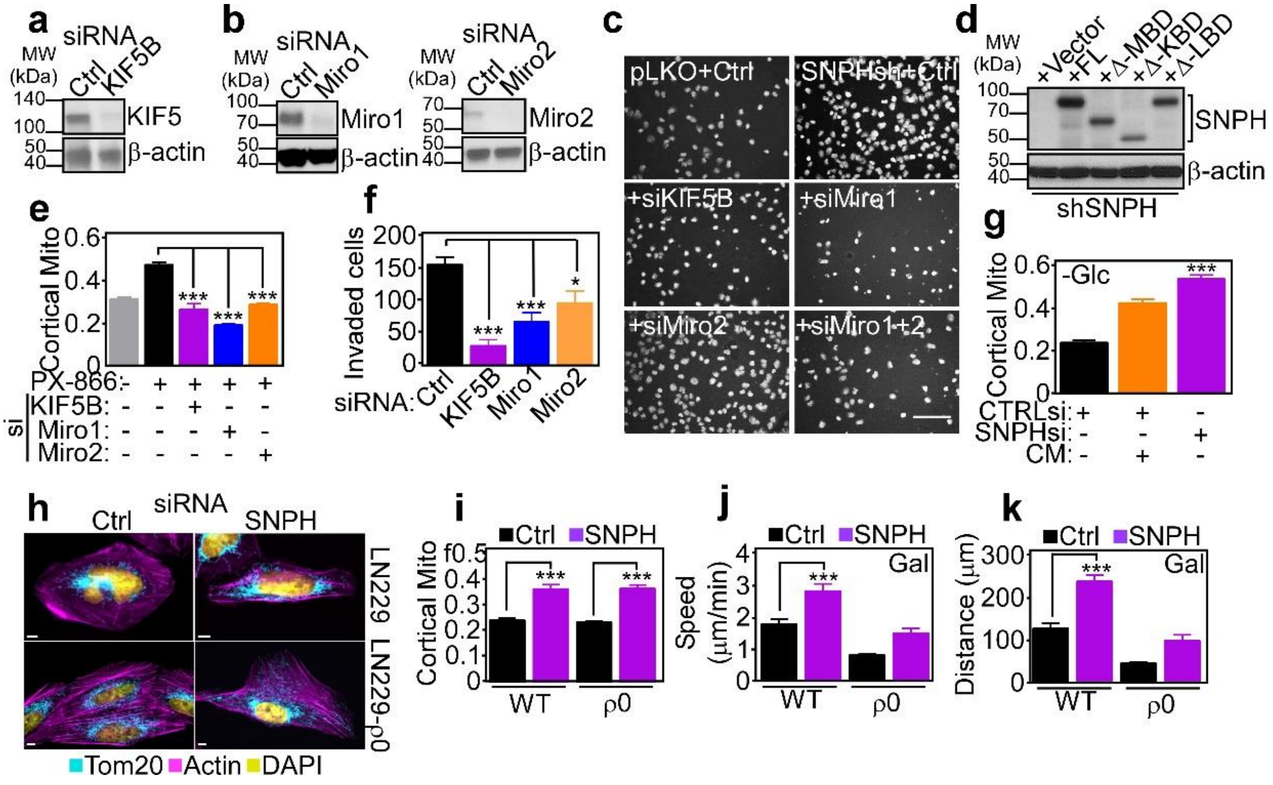

Supplementary Figure 6. **Requirements of mitochondrial trafficking by SNPH.** (**a**,**b**) PC3 cells were transfected with control (Ctrl) or KIF5B (**a**), Miro1 or Miro2 (**b**) siRNA and analyzed by Western blotting. (**c**) PC3 cells were transduced with pLKO or SNPH-directed shRNA, transfected as in (a,b) and analyzed for Matrigel invasion. DAPI-stained nuclei of invaded cells are shown. Scale bar, 200 µm. (**d**) PC3 cells were transfected with vector, full length (FL) or SNPH mutants. MTB, microtubule-binding domain; KBD, kinesin-binding domain; LBD, LC8-binding domain. (**e**) LN229 cells were transfected with siRNAs, treated with small molecule PI3K inhibitor, PX-866 (5 µM, 48 h), and analyzed by fluorescence microscopy. Quantitation of cortical mitochondria is represented as mean ± s.e.m. Sample sizes were n(-PX-866)=43, n(+PX-866)=40, n(KIF5B)=17, n(Miro1)=29, n(Miro2)=23. ***, p<0.001. (**f**) PC3 cells transfected with the indicated siRNAs were analyzed for invasion across Matrigel. Data are represented as mean ± s.e.m. (n=3). *, p<0.05; ***, p<0.001. (**g**) LN229 cells were transfected with the indicated siRNA, cultured for 24 h in glucose (Glc)-depleted medium, and mitochondrial repositioning to the cortical cytoskeleton was quantified. A positive control group was stimulated with fibroblast conditioned medium (CM). Data is represented as mean ± s.e.m. Sample sizes were n(Ctrl,-CM)=32, n(Ctrl+CM)=35, n(SNPH,-CM)=34. ***, p<0.0001. (**h**) Wild type or oxidative phosphorylation-deficient LN229 ρ0 cells were transfected with the indicated siRNA, stained and analyzed by fluorescence microscopy. Representative cells are shown. Scale bar, 5 µm. (**i**) Cortical mitochondria signal from cells in (h) was quantitated and normalized to total mitochondria fluorescence. Data is represented as mean ± s.e.m. Sample sizes were n(WT,Ctrl)=37, n(WT,SNPH)=32, n(ρ0,Ctrl)=33, n(ρ0, SNPH)=31. ***, p<0.0001. (**j**,**k**) Cells were maintained in galactose(Gal)-containing medium, transfected with control siRNA (Ctrl) or SNPH-directed siRNA, and analyzed in a 2D chemotaxis chamber with quantification of speed of cell migration (**j**) and distance traveled per cell (**k**). Data are represented as mean ± s.e.m. Sample sizes were n(WT,Ctrl)=50, n(WT,SNPH)=47, n(ρ0,Ctrl)=49, n(ρ0,SNPH)=47. ***, p=0.0005.

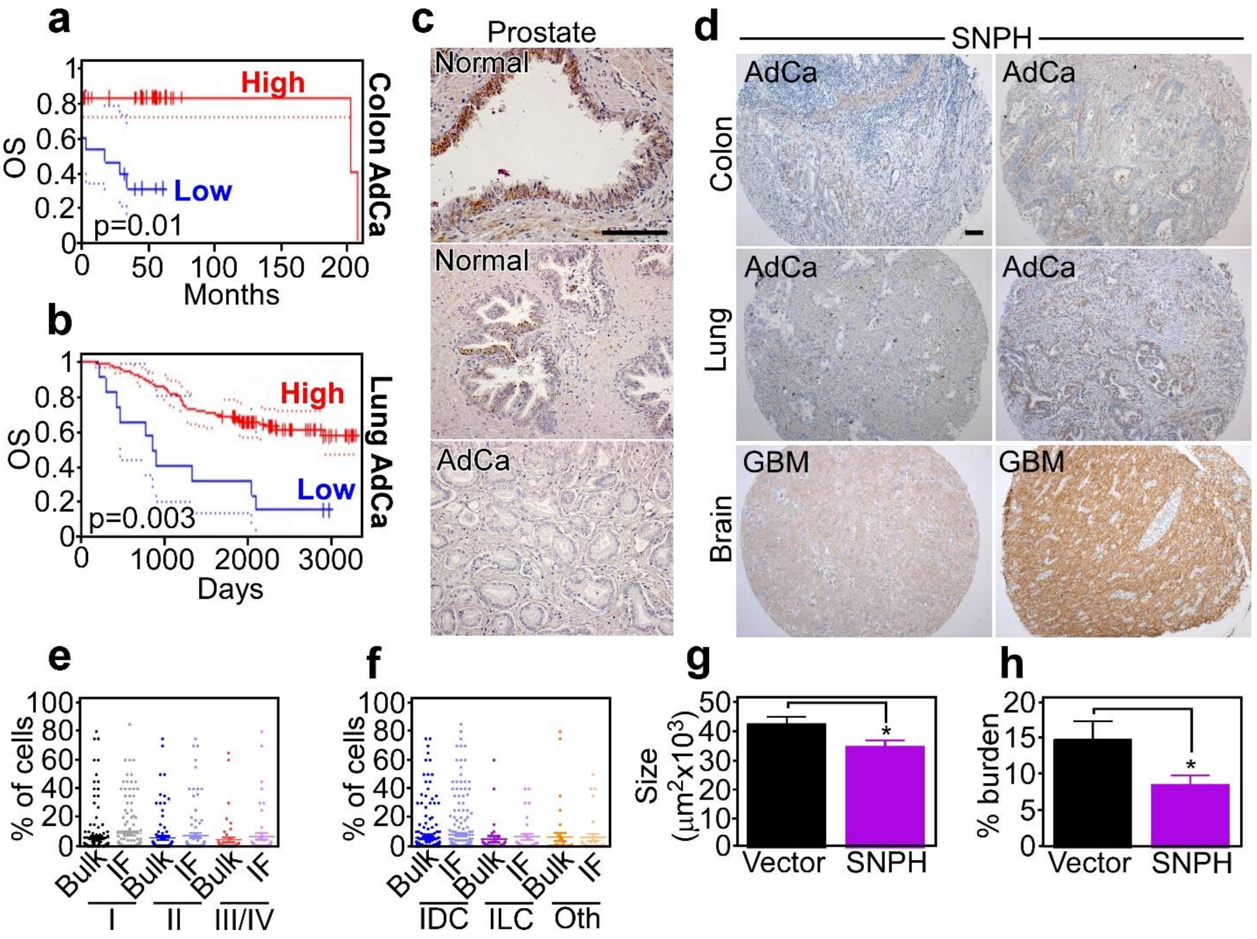

Supplementary Figure 7. **SNPH regulation of tumor progression.** (**a,b**) Overall survival (OS) of patients with diagnosis of colon adenocarcinoma (**a**) or lung adenocarcinoma (**b**) stratified for SNPH expression (High *versus* Low). Sample sizes for Colon AdCa were n(low)= 18, n(high)= 37. Sample sizes for Lung AsCa were n(low)=12 , n(high)=105. (**c,d**) Primary tissue samples representative of normal human prostate or prostatic adenocarcinoma (AdCa, **c**), or adenocarcinoma of colon or lung or glioblastoma (GBM, **d**) were stained with an antibody to SNPH by immunohistochemistry. Representative images are shown. Scale bar, 100 μm. (**e,f**) Patients with breast carcinoma were examined for SNPH expression by immunohistochemistry according to stage (**e**) or histotype (**f**). Bulk, core primary lesion; IF, invasive front; IDC, invasive ductal carcinoma; ILC, infiltrating lobular carcinoma; Oth, others. Each symbol corresponds to an individual patient. (**g,h**) PC3 cells stably transfected with vector or SNPH cDNA were injected in the spleen of immunocompromised mice and the number of liver metastatic foci (**g**) or overall metastatic burden (**h**) in reconstituted animals was quantified in serial tissue sections 500 μm apart by morphometry. Data are represented as mean ± s.e.m. (n=15).*, p=0.01-0.03.

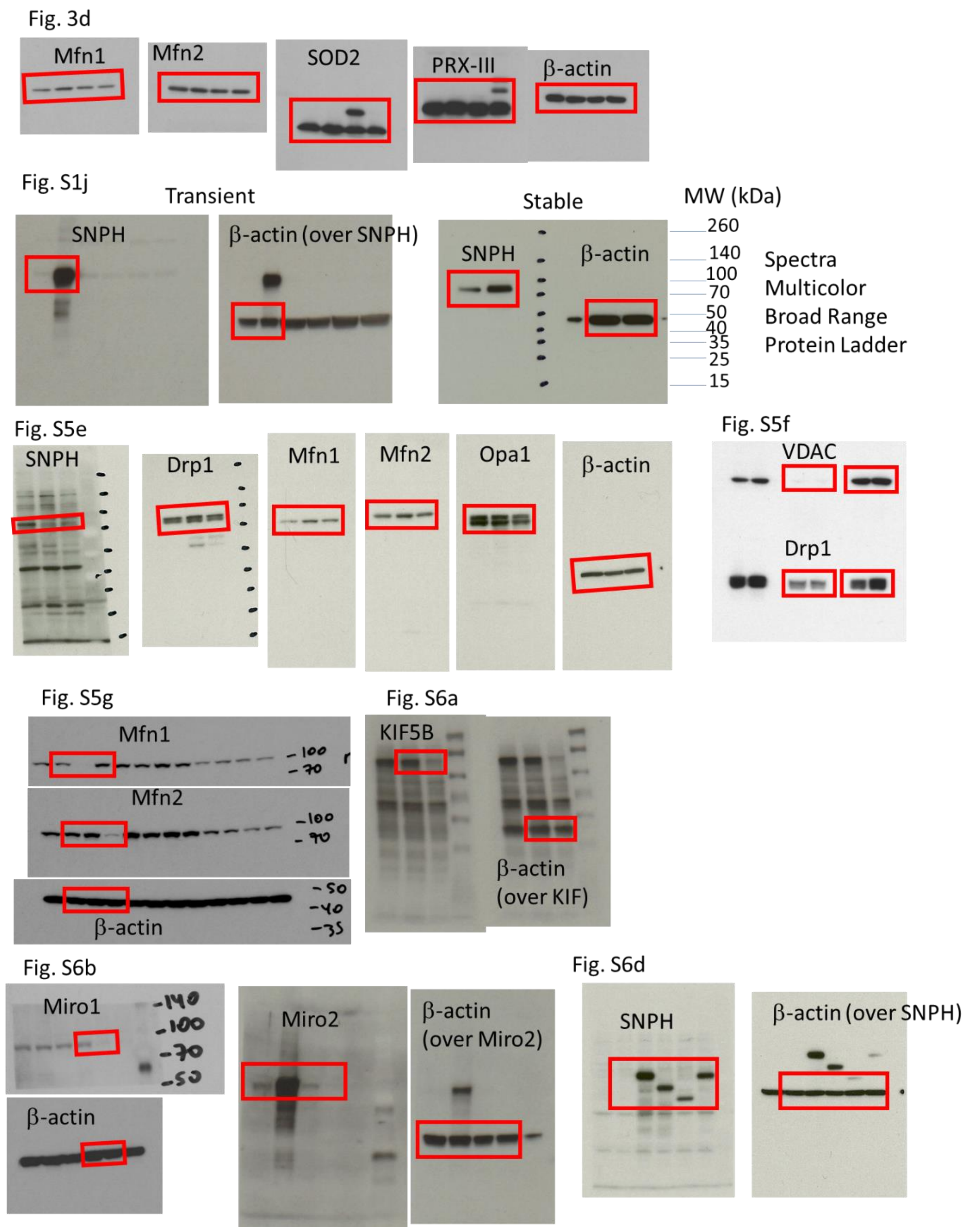

Supplementary Figure 8. WB panels from main Figures and Supplementary Figures are shown as full blots and the cropped areas are indicated in red boxes.

Supplementary Table 1. Functional categories enriched among the 174 candidate hits from sthe hRNA screening.

Group name	Source	Category	Enrich	Sensitivity	p-value	FDR
**Cytoskeleton**	SP	coiled coil	2.1	38/2019 (1%)	2E-05	0%
	MF	GO:0008092: cytoskeletal protein binding	3.5	11/504 (2%)	9E-04	1%
**Adhesion**	KEGG	hsa04510:Focal adhesion	6.3	7/201 (3%)	5E-04	0%
	KEGG	hsa04520:Adherens junction	11.8	5/77 (6%)	7E-04	1%
	SP	laminin egf-like domain	14.8	4/30 (13%)	0.002	3%
**Protein Kinase**	SP	tyrosine-specific protein kinase	16.2	7/48 (14%)	4E-06	0%
	MF	GO:0004713: protein tyrosine kinase activity	8.8	9/166 (5%)	7E-06	0%
	MF	GO:0004714: transmembrane receptor protein tyrosine kinase activity	14.5	6/67 (8%)	5E-05	0%
	SP	tyrosine-protein kinase	8.0	8/111 (7%)	6E-05	0%
	BP	GO:0007169: transmembrane receptor protein tyrosine kinase signaling pathway	5.7	9/224 (4%)	2E-04	0%
	BP	GO:0007243: protein kinase cascade	3.5	9/370 (2%)	0.004	6%
	BP	GO:0006468: protein amino acid phosphorylation	2.6	12/667 (1%)	0.006	10%
	BP	GO:0007167: enzyme linked receptor protein signaling pathway	4.2	10/342 (2%)	6E-04	1%
	SP	autophosphorylation	11.3	5/49 (10%)	1E-03	1%
**WD repeat**	SP	wd repeat	3.6	9/276 (3%)	0.003	4%
**Cell Migration**	BP	GO:0030334: regulation of cell migration	5.1	6/169 (3%)	0.006	10%

Source: SP=swiss-prot keyword, MF = molecular function from gene ontology, BP = biological process from gene ontology, KEGG = pathway from KEGG database. FDR=false discovery rate. Enrich= enrichment fold. Sensitivity follows K/N (P%) format, where K=number of genes from the list and from the category, N=total number of genes from the category, P% = percent of genes in the category that appeared in analyzed gene list.

Supplementary Table 2. Clinico-pathological characteristics of the breast cancer series analyzed for SNPH expression[1].

Histotype		IDC (n=252)	ILC (n=33)	Others (n=39)[2]
Age (Y, mean)		60	64	62
CIS[3]		22	8	10
ER[4]	pos	213	51	35
	neg	37	3	4
	na	2	-	-
PR[4]	pos	167	23	27
	neg	83	10	11
	na	2	-	-
HER2 (IHC)[5]	pos	129	2	4
	neg	185	30	34
	na	2	1	1
HER2 (amplified)	pos	4	0	0
Met LN[6]	neg	149	26	27
	pos	103	7	12
Met LN >10%[7]		54	4	4
STAGE[8]	I	124	18	21
	II	83	9	15
	III/IV	45	6	3

[1] The number of patients per category is reported. Neg, negative; pos, positive
[2] The following breast cancer histotypes are included: tubular, medullary, mucinous, papillary, mixed, signed ring.
[3] CIS, carcinoma in situ lesions detected in patients with the indicated breast cancer histotype
[4] ER, estrogen receptor; PR, Progesteron receptor
[5] HER2 immunohistochemistry (IHC) was considered positive if a 2+ or a 3+ score were assigned. Specimens with an HER2 IHC score 2+ were then analyzed for gene amplification (HER2 amplified).
[6] Met LN, metastatic lymph nodes
[7] Met LN >10%, the number of metastatic lymph nodes represented more than 10% of the analyzed LN.
[8] According to the American Joint Committee on Cancer -AJCC- TNM system
